# Investigation of chalcopyrite removal from low-grade molybdenite using response surface methodology and its effect on molybdenum trioxide morphology by roasting†

**DOI:** 10.1039/d3ra02384b

**Published:** 2023-05-15

**Authors:** Reza Behmadi, Masoud Mirzaei, M. Reza Afshar, Hamidreza Najafi

**Affiliations:** a Department of Materials Engineering, Science and Research Branch, Islamic Azad University Shohada Hesarak Blvd., Daneshgah Square, Sattari Highway Tehran 1477893855 Iran mafshar@srbiau.ac.ir; b Department of Extraction & Recycling Materials, Research and Development of Engineering Materials Research Center, Science and Research Branch, Islamic Azad University Shohada Hesarak Blvd., Daneshgah Square, Sattari Highway Tehran 1477893855 Iran; c Department of Chemistry, Faculty of Science, Ferdowsi University of Mashhad Mashhad 9177948974 Iran mirzaeesh@um.ac.ir; d Khorasan Science and Technology Park (KSTP) 12th km of Mashhad-Quchan Road Mashhad 9185173911 Khorasan Razavi Iran

## Abstract

In this research, purification of molybdenite concentrate (MoS_2_) using a nitric acid leaching process was employed for the improvement of molybdenum trioxide morphology during oxidative roasting in an air atmosphere. These experiments were performed using 19 trials designed with response surface methodology and three effective parameters being temperature, time, and acid molarity. It was found that the leaching process reduced the chalcopyrite content in the concentrate by more than 95%. The influence of chalcopyrite elimination and roasting temperature on the morphology and fiber growth of the MoO_3_ was also investigated by SEM images. Copper plays an important role in controlling the morphology of MoO_3_ and its decrease led to enhancing the length of quasi-rectangular microfibers from less than 30 μm for impure MoO_3_ up to several centimeters for purified MoO_3_.

## Abstract 2

Chalcopyrite removal to enhance the length of MoO_3_ fibers.

## Graphical abstract

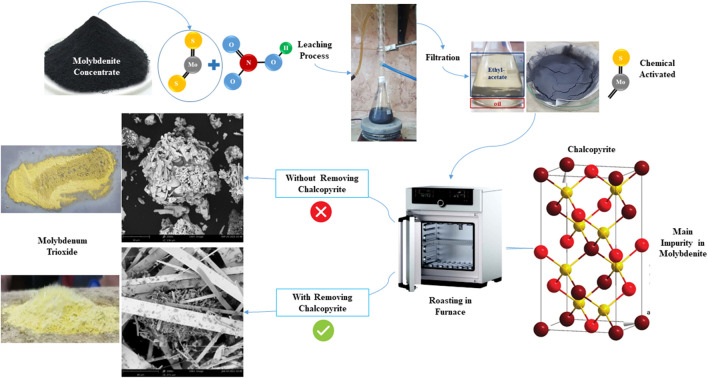

## 1. Introduction

Molybdenite concentrate in the form of molybdenum disulfide is one of the most important mineral sources for producing molybdenum and its compounds.^1^ This mineral-based substance is a by-product in copper mines and is obtained *via* froth flotation processes.^2^ The crude material is converted into technical grade molybdenum trioxide by oxidative roasting in an air atmosphere in the presence of sodium chloride, lime or soda ash and is followed by purification by chemical leaching.^3–5^ Copper is typically found in nature as a sulfide, oxide, carbonate, or silicate mineral.^6^ The most significant copper sulfide mineral is chalcopyrite.^7,8^ Sulfide minerals like galena (PbS), pyrite (FeS_2_), and sphalerite (ZnS) are frequently found together with it.^9^ Chalcopyrite, a hard-to-leach mineral in acidic solutions, is typically the primary copper impurity in molybdenite concentrates.^10,11^ In leaching processes, secondary copper sulfides like covellite (CuS) and chalcocite (Cu_2_S) dissolve more quickly than chalcopyrite.^12–14^ A molybdenite concentrate's market value drastically decreases when chalcopyrite is present. To meet consumer requirements, molybdenite concentrate should contain less than 0.5 percent copper.^5^ When copper is present in the form of chalcopyrite, removing it from molybdenite by leaching necessitates the use of extremely aggressive solutions.^13,15,16^ Flotation processes frequently is used high doses of sodium sulfide and sodium cyanide to remove copper from molybdenite concentrates in industrial sectors.^5,17^

Much effort has been applied to copper removal and molybdenum extraction from molybdenite concentrate.^18–21^ Copper in the form of chalcopyrite (CuFeS_2_) is one of the most important impurities in molybdenite concentrates.^22^ Using nitric acid as a solvent and an oxidant, or using sodium dichromate, sodium chlorate, and hypochlorite as oxidants, leaching of molybdenite has been investigated by some researchers.^4^ Padilla *et al.*^13,23^ used both sulfidation and leaching methods to decrease the chalcopyrite content and observed a selective elimination for copper and minimum molybdenum dissolution in a mixed solution of H_2_SO_4_–NaCl–O_2_ with acceptable efficiency of around 96%.^13,23^ Agaçayak *et al.*^9^ obtained optimum leaching conditions at a temperature of 80 °C with a solid-to-liquid ratio of 1/500 g/mL, 4 M HNO_3_, and rotation speed of 400 rpm for 3 h. The extraction of copper from chalcopyrite concentrate in nitric acid solution was approximately 80% under these conditions.^9^ Smirnov *et al.*^24^ addressed copper removal from molybdenite concentrate under high pressure conditions using sulfuric acid and nitric acid as the main medium and catalytic additive, respectively. They found that using nitric acid considerably increases the rate of molybdenite oxidation and subsequently reduces some operating conditions like temperature and pressure.^24^ Goodarzi *et al.*^4^ applied the analysis of variance (ANOVA) with the Taguchi method on the extraction of molybdenum from its concentrate and studied various effective parameters including reaction time, reaction temperature, hydrogen peroxide concentration, sulfuric acid concentration, pulp density and rotation speed. Based on their findings, the most important factors were H_2_O_2_ concentration, pulp density, and reaction temperature with reaction time being of marginal significance and rotation speed and H_2_SO_4_ concentration not statistically significant factors.^4^ On the other hand, Benzeşik *et al.*^25^ investigated the effects of H_2_SO_4_ concentration and the solid-to-liquid ratio on removal of copper from molybdenite concentrate and showed that 0.6 M H_2_SO_4_ can remove Cu at contents of 2.56 wt% up to 95.92% efficiency.^25^ Petrovic *et al.*^26^ studied leaching the chalcopyrite concentrate *via* HCl acid and H_2_O_2_ as an acid medium and strong oxidizer solution, respectively. Their experiments showed that 33% of the copper was dissolved in the first 60 min of a three-hour reaction.^26^ Almeida *et al.*^27^ studied the dissolution of chalcopyrite in acid solutions like nitric (HNO_3_), sulfuric (H_2_SO_4_), and hydrochloric (HCl) acid using electrochemical impedance spectroscopy (EIS) and found that dissolution of chalcopyrite is controlled by diffusion.^27^ Medvedev *et al.*^28^ carried out a series of kinetic studies on leaching molybdenite concentrate by investigating the effect of temperature and nitric acid concentration. Their suggested model has extracted molybdenum from the molybdenite concentrate up to 95%. Shalchian *et al.*^29^ addressed the effect of mechanical activation parameters on the leaching rate of molybdenite concentrate in nitric acid solution by ANOVA to determine the statistics of the main variations such as activation time, ball-to-powder ratio and rotation speed. They found that mechanical activation enhances the molybdenite leaching rate, considerably.^29^

Technical grade molybdenum trioxide (MoO_3_) is one of the initial substances used in a variety of industrial applications such as the production of molybdenum metal, ferromolybdenum alloy,^3^ batteries, sensors and electronic display devices, field emission, gas-chromic, photo-chromism devices, as well as the development of catalysts for petroleum and petrochemical industries because of its catalytic properties.^30,31^ Roasting is frequently used in the production of molybdenum trioxide from molybdenite concentrate.^32,33^ Some methods that are used for the preparation of molybdenum trioxide with different morphologies include hydrothermal/solvothermal methods, thermal evaporation, a sonochemical process, mechanical grinding and sonication, electro-spinning, and nanosecond-duration plasma discharges techniques.^30,34,35^ The temperature of the roasting process is usually between 400 to 700 °C in an air atmosphere. Some studies have shown that the oxidation rate is slow up to 444 °C and then increases up to 580 °C while becoming almost constant at 640 °C.^32^ Kim *et al.*^3^ found that during the oxidative roasting of low-grade molybdenite concentrate, more than 95% of the particles changed into molybdenum trioxide at 550 °C after 40 min.^3^ Basically, the thermodynamically stable orthorhombic MoO_3_ (α-type), metastable monoclinic MoO_3_ (β-type), and hexagonal MoO_3_ (h-type) are common polymorphic phases of molybdenum trioxide.^36,37^ Morphologically, depending on the starting materials and preparation methods, the shapes of molybdenum trioxide particles include belts, rods, tubes, fibers, wires, spheres, flakes, among others with different sizes from nano-to micrometers.^30,31^ Sui *et al.*^30^ synthesized α-MoO_3_ with a hierarchical flower-like structure using a facile solvothermal method and followed by calcination at 400 °C. The diameter of the microrods obtained was around 3–5 μm with average diameters of 150–200 nm.^30^ Szkoda *et al.*^38^ investigated the influence of water treatment on the morphology and crystalline structure of MoO_3_. Their method was based on the electrochemical anodization of a Mo plate inside the fluorine solution. Annealing at 500–700 °C created the regular MoO_3_ structure with a length of about 1 μm.^38^ Ornelas *et al.*^39^ synthesized molybdenum trioxide by a microwave assisted hydrothermal method and changed the morphology of MoO_3_ hexagonal rods from 3D to 2D using external heat treatment in an air atmosphere at a temperature above 650 °C.^39^

From the point of view of impurity effects on molybdenum trioxide morphology, it has been discovered by some researchers that reducing the iron amount with leaching provides thinner fibers during the process of oxidation. Overall, these studies express that the removal of iron from molybdenite can have a significant impact on the morphology of molybdenum trioxide fibers, resulting in smaller and more uniform fibers.^40–42^

In the present work, to investigate the influence of chalcopyrite on the molybdenum trioxide morphology after roasting the molybdenite concentrate at different temperatures, a series of nitric acid leaching experiments were designed with temperature (°C), time (h), and acid molarity (M) as the primary variables.

## 2. Experimental

### 2.1. Materials

A sample of raw molybdenite concentrate (RMC), a low-grade ore containing 52.38% Mo, 35.01% S, 2.45% Cu, 4.13% Fe and 6.03% other elements, was supplied from the Sarcheshmeh Copper Complex mine in the Kerman province of Iran. Ethyl acetate and nitric acid 65% (HNO_3_) were purchased from Sigma-Aldrich. The chemical composition of the RMC was analyzed by the ICP-MS method.

### 2.2. Washing and leaching process

Removal of the residual flotation oil in the RMC was carried out using several washing steps with ethyl acetate and distilled water. Subsequently, the washed molybdenite concentrate (WMC) was filtered under vacuum on Whatman 42 filter paper and dried at 105 °C for 6 h. In the next step, 32 g of WMC was added to 800 mL of either 0.3, 0.6 and 0.6 M nitric acid solution in a 1000 mL Erlenmeyer flask and mixed completely with a magnetic stirrer for times of 1, 2, or 3 h) at 70, 80, and 90 °C (first value of each variable used in run 1. Subsequently, filtration of the purified molybdenite (PMC) was performed under vacuum followed by drying at 105 °C for 6 h.

### 2.3. Roasting process

Technical molybdenum trioxide (α-MoO_3_) fibers were obtained *via* a roasting method. In order to find the optimum conditions for the roasting process, chemically activated pure MoS_2_ (PMS) was heated in three series of experiments at 600 °C, 625 °C, and 650 °C for 1 h each.

### 2.4. Characterization

A scanning electron microscope (SEM) proX model with a voltage of 15 kV was used for the analysis of the sample morphology. A Thermo Scientific ELEMENT 2XR high-resolution inductively coupled plasma-mass spectrometer (ICP-MS) and the inductively coupled plasma-optical emission spectrometry (ICP-OES) equipped by a spectrometer with axially viewed plasma were used to determine the metal contents in molybdenite and acidic leaching solutions, respectively. The composition of the crystalline structure and phase content of all samples were analyzed by an XRD diffractometer (Philips X-pert Pro X-ray diffractometer). The target and wavelength were Cu Kα and 1.54060 Å, respectively. The XRD measurements were performed with a source power of 40 kV and with a 300 mA current, the scanning range was between 5° and 60° with a step size of 0.05°.

## 3. Results and discussions

**Fig. 1:**
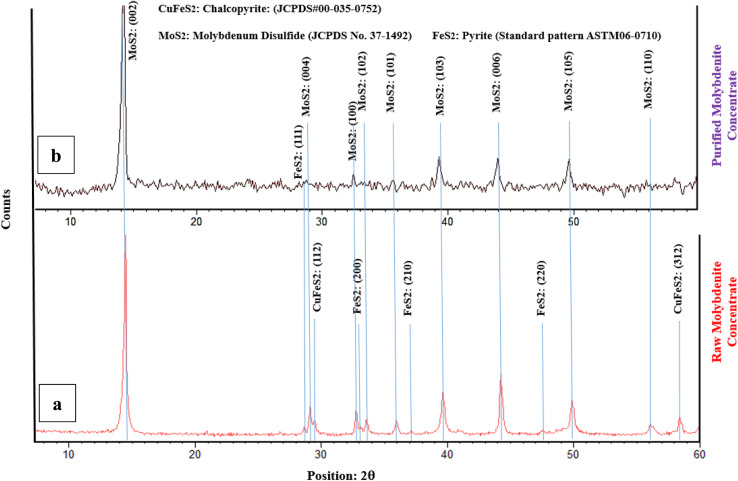
The XRD graph of raw and purified molybdenite concentrate.

According to the chemical composition of RMC, copper and iron are the main impurities in the raw molybdenite concentrate. Fig. 1 shows the XRD graph to identify the minerals present in the initial ore and purified concentrate with nitric acid. Mineralogical study of the sample showed molybdenite (MoS_2_) as the main mineral phase and chalcopyrite (CuFeS_2_) and pyrite (FeS_2_) as the minor mineral impurities present. Also, many low intensity peaks were found which correspond to sulfide compounds such as FeS_2_ and CuFeS_2_.^26,43^ However, it was very difficult in the study to identify such sulfide compounds at the sample. This might be the reason that the amounts of all such sulfide compounds contained in the samples are relatively small.^3^

The crystalline MoS_2_ structure was indexed at 14.4°, 28.4°, 35.9°, 39.6° and 43.3° corresponding to the (002), (004), (102), (103) and (006) crystal planes, which matched with the corresponding standard card (JCPDS card No. 75-1539) of the hexagonal MoS_2_ structure. The strongest diffraction peak is indexed to the (002) plane of MoS_2_, which means that a large number of grains are in parallel orientation, which is in good agreement with the literature.^25,28,44^ The peak at 2*θ* = 14.4° is an indication of the sulphur content of the concentrate.^32,45^

The XRD pattern for chalcopyrite in Fig. 1a, as the standard card of JCPDS# 00-035-0752, has peak positions of 29.6° and 58.5°, which correspond to the (112) and (312) crystallographic planes, respectively. The successful purification of the molybdenite concentrate through the HNO_3_ leaching process is verified by the absence of a chalcopyrite peak in Fig. 1b.

Additionally, the X-ray diffraction (XRD) pattern for pyrite present in molybdenite may vary depending on the specific sample and the experimental conditions used in the study. The standard card of ASTM06-0710 indicates that characteristic peaks for pyrite are typically observed at around 28.7°, 33.1°, 37.2°, and 47.6°, which correspond to the (111) (Fig. 1b), (200), (210), and (220) (Fig. 1a) crystallographic planes, respectively. The XRD results demonstrate a considerable decrease in the pyrite content following the leaching process.

The purpose of the present study was to identify the influence of roasting temperature and copper elimination on the molybdenum trioxide morphology. To investigate the influence of the copper presence or absence on technical molybdenum trioxide morphology during thermal treatment and according to the initial chemical composition of the molybdenite concentrate, removal of CuFeS_2_ was typically selected as the main purpose of the leaching process, because it is recognized as one of the most important impurities in the ore. Hence, to obtain the most efficient conditions for copper removal, optimization of the leaching process was determined by Central Composite Design (CCD).^46–49^ In the leaching process, three important factors affect copper removal and consequently the catalytic properties of molybdenite; the molarity of nitric acid, the temperature, and the leaching time.

By applying the multivariate Response Surface Methodology (RSM), the optimization of the process and the design of experiments were conducted faster, more economically, and more accurately.^50^ The molarity of nitric acid, temperature, and leaching time was regarded as independent variables; the residual copper was taken as the response variable. In all experiments the rotation speed and pulp density (powder molybdenite/nitric acid solution volume ratio) were 400 rpm and 1 : 6, respectively. The quadratic model of (eqn (1)) was used to explain the relationship of responses as a function of independent variables:^51–53^1
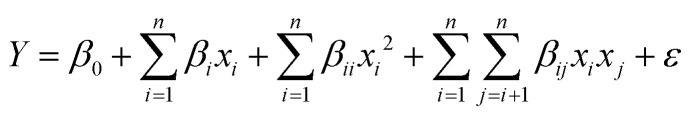
where *Y* is the response mean copper removal efficiency, *β*_0_ is a constant coefficient, also *β*_*i*_, *β*_*ii*_, and *β*_*ij*_ denote the coefficients of linear, quadratic, and dual interaction effects. *x*_*i*_ and *x*_*j*_ are the independent variables and *ε* indicates the error of the model. Table 1 presents the leaching data obtained from the purification of molybdenite concentrate. To obtain information on the reproducibility of the experiment, five replicated center points were included. This design with a total of 19 experiments enabled a quadratic model equation. Model terms that showed low significance on the results could be excluded to identify the parameters with the main effect on the responses. However, every single term was included to take significant linear interactions between them into account, even though they had a minor influence on the results. From these results, the optimum working conditions were chosen as a 0.6 M HNO_3_ solution at 80 °C for 2 h, to remove CuFeS_2_.

**Table 1:** Experimental design *via* design-expert software based on central composite design (CCD) technique with leaching efficiency values (ICP-MS)

Run	Independent variables			Response	
	Nitric acid (molarity)	Time (hour)	Temperature (°C)	Residual Cu (ppm)	Cu removal efficiency (%)
1	0.3	3	70	6239	74.53
2	0.6	2	80	1295	94.71
3	0.3	3	90	2903	88.15
4	0.3	2	80	1411	94.24
5	0.6	2	80	2709	88.9
6	0.3	1	70	12 656	48.34
7	0.9	2	80	2035	91.69
**8**	**0.6**	**2**	**80**	**1039**	**95.75**
9	0.6	2	70	3470	85.83
10	0.9	1	70	2517	89.72
11	0.3	1	90	8460	65.46
12	0.9	1	90	749	96.94
13	0.6	2	80	1750	92.85
14	0.9	3	70	718	97.06
15	0.6	2	80	1896	92.26
16	0.6	3	80	835	96.59
17	0.6	1	80	1935	92.10
18	0.6	2	90	1662	93.21
19	0.9	3	90	1492	93.91

RSM is one of the most efficient statistical and mathematical methods that is used for the investigation of multiple factors.^54^ In this work, the CCD was used to determine the effect of operating parameters (the molarity of nitric acid, working temperature, and leaching time) on the efficiency of copper removal. In the model equation obtained, the efficiency of copper removal (*η*) was determined as a function of (*A*) acid solution concentration, (*B*) operating temperature (*C*), and leaching time (eqn (2)). The model that relates the responses to operating parameters was developed using RSM in terms of actual factors:

2 *η* = 9.86*A* + 4.22*B* + 5.77*C* − 9.92*B*^2^ − 5.57*AC* + 93.23

Table 2 presented ANOVA results to determine the statistical significance of the major variable effects as well as the interaction effects. The significance level employed in the analysis evaluated by *p*-values is less than 0.05.^55^ The significance of the predicted models and their parameters can be determined using ANOVA.^56^ The model *F*-value and *p*-value (prob > *F*) are 9.72 and 0.0005, respectively, which implies the model is significant.^57^ Generally, the larger *F*-statistic and smaller *P*-value indicate a higher significance of the corresponding variables. More specifically, values of “prob > *F*” less than 0.05 indicates that model terms are significant, while values greater than 0.1 are not considered significant items. The *p*-value is less than the significance level, so the sample data provide sufficient evidence that the developed regression model fits the data better than the model with no independent variables.^58^

**Table 2:** Obtained results of ANOVA for leaching molybdenite concentrate for copper removal^a^

Factor	Sum of squares	DOF	Mean square	*F*-Value	*P*-Value
Model	2197	5	439.42	9.72	0.0005
*A*: acid concentration	972	1	972.20	21.50	0.0005
*B*: temperature	178	1	178.00	3.94	0.0688
*C*: time	332	1	332.70	7.36	0.0178
*AC*	248	1	248.31	5.49	0.0357
*B* ^2^	465	1	465.87	10.30	0.0068
Residual	587.81	13	45.22	—	—
Pure error	27.81	4	6.95	—	—
Cor total	2784.89	18	—	—	—

a *R* ^2^ = 0.7889, adjusted *R*^2^ = 0.7077, predicted *R*^2^ = 0.4561, adequate precision = 11.960.

By considering this criterion, the terms *A*, *C*, *AC* and, *B*^2^ for which corresponding *p*-values are lower than 0.05, can be considered significant and effective model parameters. The values of *R*^2^, adjusted *R*^2^ and predicted *R*^2^ for the first model (*η*) are 0.7889, 0.7077 and 0.4561, respectively. These values indicate that the model fitting is appropriate and accurate. Adequate precision which measures the signal to noise is desirable at ratios greater than 4. In this case, it is 11.960, which indicates the experimental data can be predicted accurately by the model. R-squared measures the strength of the relationship between the model and the dependent variable.^59^ The *F*-test of overall significance is the hypothesis test for this relationship. Because the overall *F*-test is significant, it can be concluded that R-squared does not equal zero, and the correlation between the model and dependent variable is statistically significant.^60^

**Fig. 2:**
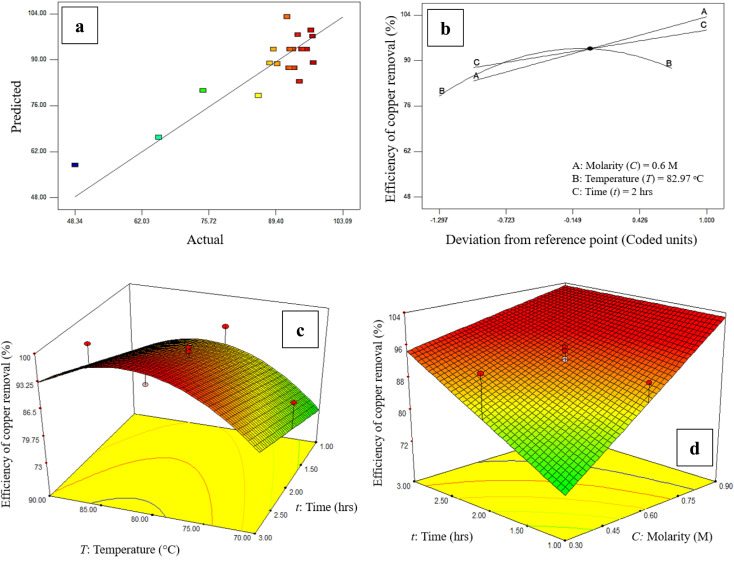
The effect of studied parameters on copper removal efficiency: (a) predicted *versus* actual efficiency data for leaching of molybdenite concentrate. Perturbation curves for assessing of main factors on the efficiency of copper removal, (b) actual factors consist of molarity (*A*) = 0.6 M, temperature (*B*) = 82.97 °C, and time (*C*) = 2 h. (c) Leaching time and process temperature, (d) leaching time and acid concentration.

In addition, the values calculated by eqn (2), which are called predicted values, *versus* the actual values obtained by experiments, are shown in Fig. 2a which shows there is a good agreement between the predicted values and the actual ones. Three-dimensional response plots which were used to investigate the existence of a relationship between the acid media molarity and leaching time with copper removal efficiency are shown in Fig. 2c and d. The copper removal efficiency increased with both the rise of acid molarity and operating time and from Fig. 2b, the optimum operating temperature of the leaching process for removing copper in the form of chalcopyrite is around 82 °C. As a result, by increasing the contact time between the acidic solution and the MoS_2_ concentrate, both CuFeS_2_ solubility in acidic solution and the purity of MoS_2_. A limiting factor is the pulp density which, if too high means that the acid media becomes saturated with impurities faster, reducing the ability of the nitric acid to remove impurities.

Fig. 2b shows the perturbation plots related to the effect of the main factors on the efficiency of copper removal from WMC, which include the molarity of the acid solution, the operating temperature, and also the time of leaching an increase of the acid solution molarity and duration time of the leaching leads to a higher efficiency of the process. While temperatures up to 82.97 °C increases efficiency, at higher temperatures the amount of removed copper decreases to around 85%. From these experiments, run 12 (from Table 1) was selected as the optimum for the purification process of molybdenite concentrate (*T*: 80 °C, *t*: 2 h, *M*: 0.6) with an efficiency of copper removal of more than 95%.

Table 3 shows the leaching data obtained from filtered acidic solution after the purification of molybdenite concentrate. To obtain accurate data, each experiment was repeated twice. Every series of experiments was performed based on one fixed parameter and two variable parameters. Finally, the efficiency of decreasing main impurities included copper and iron is listed in Table 3.

**Table 3:** Analysis results and leaching efficiency values on filtered acidic solution (ICP-OES)

Operating parameters		Residual Cu (ppm)	Efficiency of copper removal	Residual Fe (ppm)	Efficiency of iron removal
Molarity of nitric acid	0.6	7689	78.46	3938	23.84
	0.9	8519	86.93	5601	33.91
	0.3	4859	49.59	4001	24.22
	0.6	6539	66.73	6890	41.71
	0.9	8010	81.73	5552	33.61
	0.3	3028	30.91	3226	19.53
Time of leaching	2	6111	62.34	10 817	65.48
	1	5302	54.08	7739	46.85
	3	8263	84.28	6214	37.62
	1	6027	61.53	7790	47.16
	3	8796	89.79	9629	58.29
	2	8159	83.36	10 604	64.19
Temperature of leaching	80	8550	87.24	4926	29.82
	80	8691	88.67	8352	50.56
	90	8378	85.51	8228	49.81
	90	6909	70.51	8230	49.82
	70	5919	60.4	6426	38.9
	70	5647	57.65	8428	51.02

### 3.1. Effect of nitric acid concentration

**Fig. 3:**
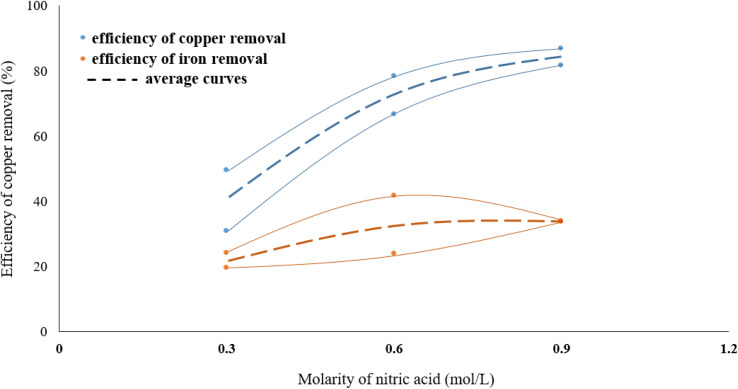
The effect of HNO_3_ molarity on removing the copper.

The effect of acid molarity on the dissolution of copper in the form of chalcopyrite was investigated using three different HNO_3_ concentrations (0.3, 0.6 and 0.9 mol L^−1^). In these experiments, the reaction temperature was kept constant at 70 °C, the solid/liquid ratio at 40 g L^−1^ and stirring speed at 500 rpm. The results, as shown in Fig. 3, demonstrate that the Cu removal increases as acid concentration increases. The molarity of nitric acid has an important effect on the copper dissolution efficiency from molybdenite concentrate. As seen in Fig. 2, leaching in 0.9 M HNO_3_ dissolved around three times as much copper from the molybdenite concentrate than a leaching in 0.3 M acid solution. This large effect of molarity on the efficiency of copper dissolution suggests that the dissolution reaction is controlled by heating.

In the range of the acid molarity studied, the dissolution rate of iron is much lower than copper and increasing the molarity of nitric acid did not have a significant effect on decreasing the iron content. On the other hand, the removal efficiency of copper is between 1.5 and 2 times higher than iron.

### 3.2. Effect of leaching time

**Fig. 4:**
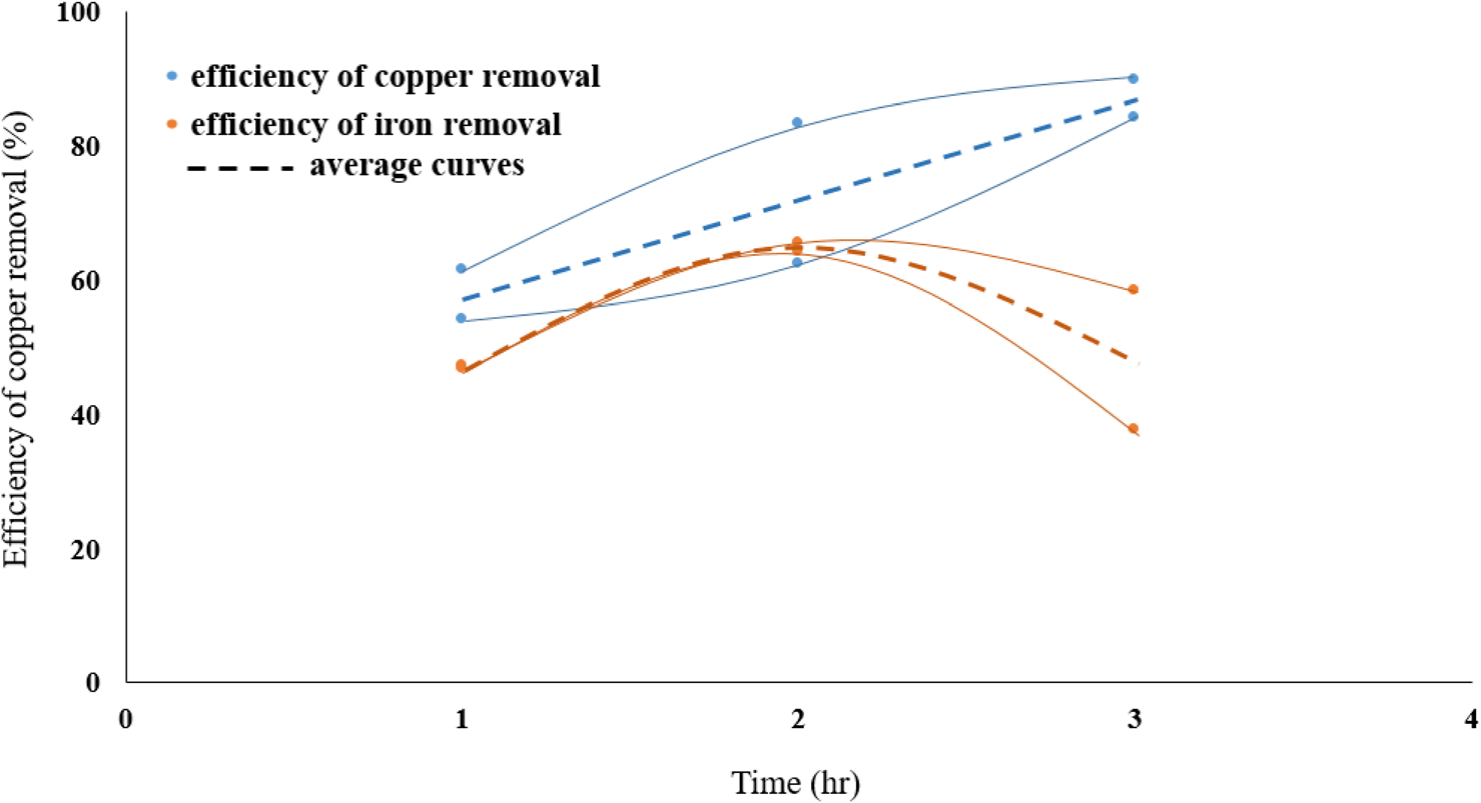
The effect of leaching time on removing the copper.

Fig. 4 presents the influence of leaching time on eliminating copper from molybdenite concentrate. The role of time on the dissolution of copper was investigated at various times (1, 2 and 3 h). In these experiments, the reaction temperature was kept constant at 70 °C, solid/liquid ratio at 40 g L^−1^, nitric acid molarity at 0.3 M, and stirring speed at 500 rpm. According to Fig. 2, the reaction rate has increased with increasing temperature. It can be concluded that chalcopyrite particles are slowly dissolved in the leach solution.

In the range of time studied and after two hours of leaching process, the dissolution of iron decreases, while increasing the exposure time of molybdenite to nitric acid enhances the dissolution efficiency of copper.

### 3.3. Effect of temperature

**Fig. 5:**
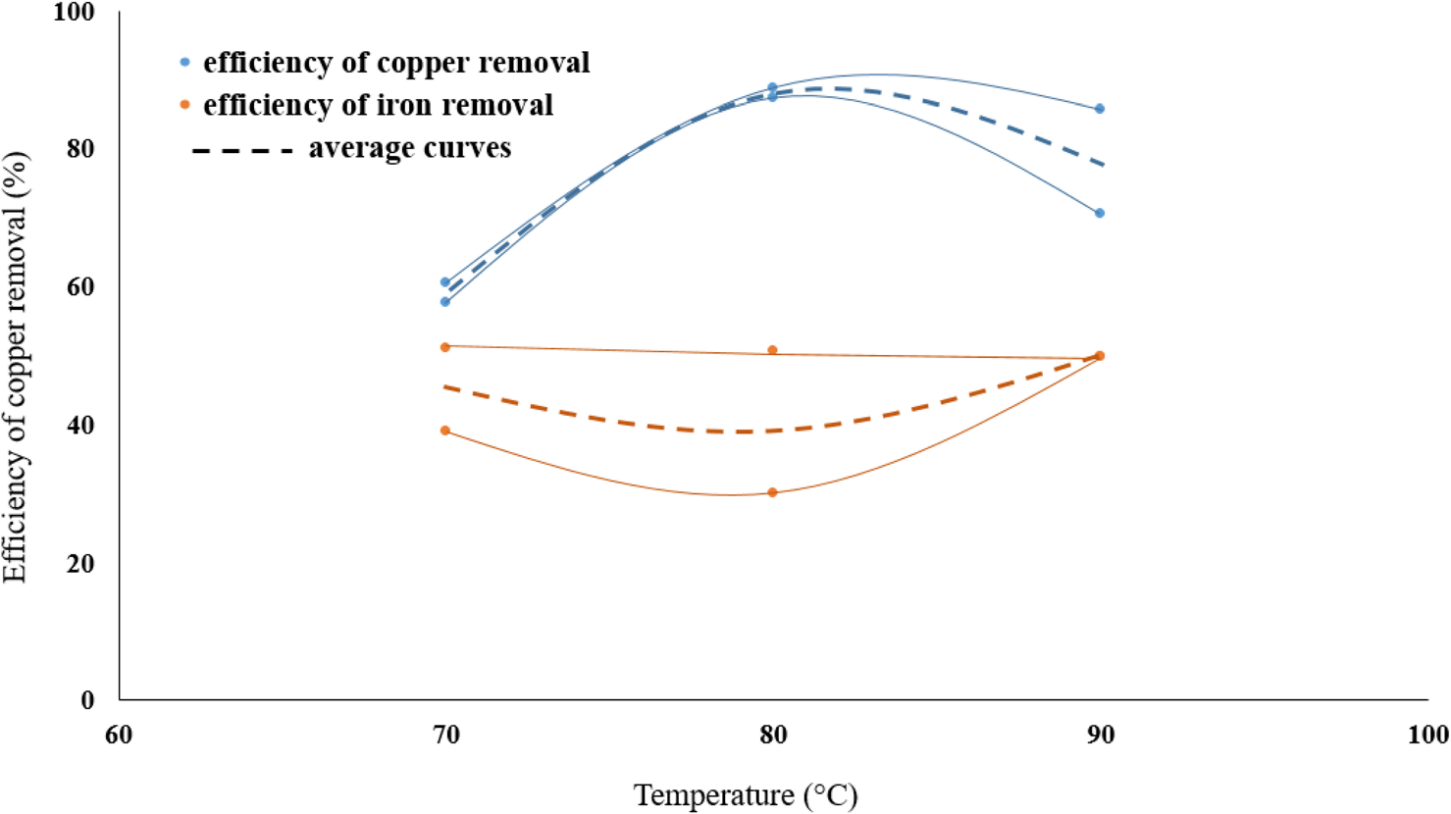
The effect of leaching temperature on removing the copper.

The effect of temperature on copper removal efficiency was investigated at 70, 80, and 90 °C. During the test, HNO_3_ concentration (0.3 M), stirring speed (500 rpm), leaching time (1 hour) and pulp density (32 g of WMC to 800 mL of acid solution) were kept constant. The results are presented in Fig. 5 which indicate that raising the temperature increases the Cu extraction but temperatures higher than 80 °C did not further increase the dissolution of copper from the molybdenite concentrate although it did have a positive effect on the reduction of iron content.

**Fig. 6:**
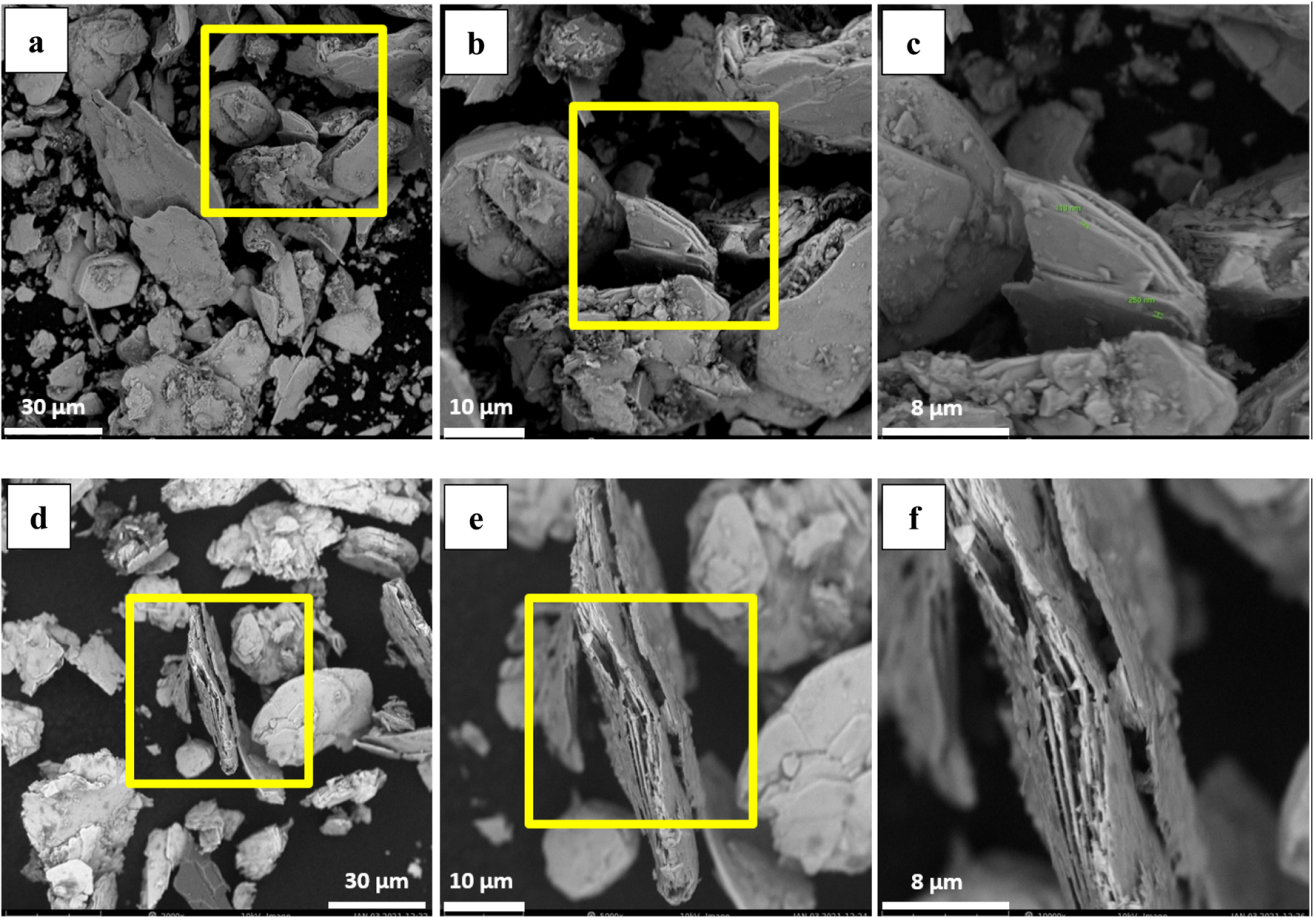
SEM image of (a–c) molybdenite concentrate washed with ethyl acetate and distilled water, (d–f) purified molybdenite concentrate by 0.6 M nitric acid at ∼82 °C for 2 h, with different magnifications (a and d) 500×, (b and e) 2000×, (c and f) 100 00×.

**Fig. 7:**
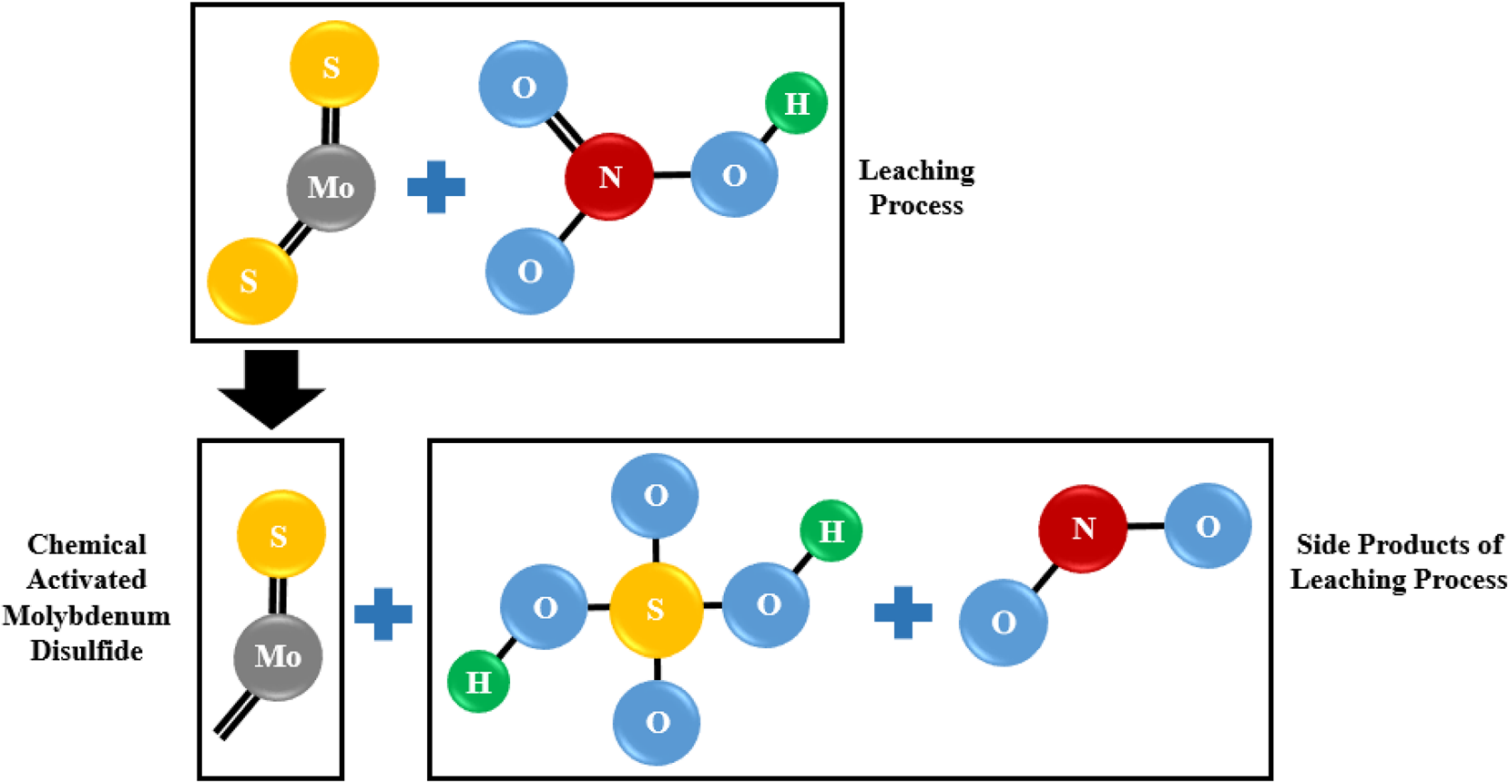
The scheme of sulfur vacancies created in MoS_2_ during the leaching process as a consequence of the reaction between molybdenum disulfide and nitric acid.

One Mo and two S atoms form strong covalent connections within MoS_2_ layers, whereas weak van der Waals forces connect MoS_2_ monolayers.^61^ The multilayered nature of particles is demonstrated by SEM images of washed MoS_2_ (WMC) (Fig. 6a–c). In treated MoS_2_, an increase in the inter-layer space is observed following leaching (Fig. 6d–f). The removal of NO_*x*_ may also have contributed to increasing the lattice expansion between MoS_2_ layers. As shown schematically in Fig. 7, and according to reaction (3) (the main reaction of sulfide minerals with acidic solutions) and reaction (4), sulfur vacancies in molybdenum disulfide can result from the partial removal of sulfur during the leaching process.^24,62^

3 3MS + 2HNO_3_ + 3H_2_SO_4_ → 3MSO_4_ + 3S^0^ + 2NO + 4H_2_O

4 MoS_2_ + 6HNO_3_ → H_2_MoO_4_ + 2H_2_SO_4_ + 6NO

Therefore, not only pyrite (reaction (5)) and chalcopyrite (reaction (6)) are oxidized, but also the separation of sulfur from MoS_2_ is caused by a chemical reaction that releases compounds like sulfuric acid (H_2_SO_4_) and nitrogen oxide (NO_*x*_).^24^

5 2FeS_2_ + 10HNO_3_ → Fe_2_(SO_4_)_3_ + H_2_SO_4_ + 10NO + 4H_2_O

As see in reaction (5), nitric acid converts pyrite to ferric sulfate (Fe_2_(SO_4_)_3_) while producing the sulfuric acid (H_2_SO_4_), nitrogen monoxide gas (NO) and water (H_2_O) are as byproducts of the reaction.

6 CuFeS_2_ + 4HNO_3_ → Cu(NO_3_)_2_ + Fe(NO_3_)_3_ + 2S + 2NO + 2H_2_O

In reaction (6), nitric acid acts as an oxidizing agent and oxidizes the sulfide ions in chalcopyrite to elemental sulfur (S). The copper and iron ions are then dissolved in the nitric acid to form copper nitrate (Cu(NO_3_)_2_) and iron nitrate (Fe(NO_3_)_3_). The nitrogen monoxide gas (NO) and water (H_2_O) are also produced as byproducts of the reaction. The sulfuric acid generated from the reaction (5) aids in speeding up the dissolution of chalcopyrite in nitric acid. So, it leads to a higher solubility of chalcopyrite in comparison to pyrite, as observed in the results obtained under same operating conditions. Hence, reaction (7) is a better representation of what occurs in reality.

7 6CuFeS_2_ + 34HNO_3_ + 3H_2_SO_4_ → 3Fe_2_(SO4)_3_ + 6CuSO_4_ + 34NO + 20H_2_O

The leaching process involves treating the molybdenite with a chemical solution, which dissolves the copper and leaves behind the molybdenite.^63^ The removal of copper from molybdenite through leaching can have a significant impact on the catalytic properties of the mineral. Molybdenite is commonly used as a catalyst in the petrochemical industry, and the presence of copper can interfere with its catalytic activity.^64^ By removing the copper through leaching, the catalytic properties of the molybdenite can be improved. The resulting material can have a higher surface area, which can increase its catalytic activity. Additionally, the removal of copper can reduce the formation of unwanted byproducts during catalysis, leading to a more efficient and selective process. On the other hand, drawbacks of unsupported MoS_2_ monolayers as catalyst include a low production rate, low porosity and a high aggregation degree, low catalytic. Thus, multilayer-MoS_2_ based catalysts can feature an enlarged active surface and have a higher activity. Thus, few-layer ordered and supported MoS_2_ catalysts are expected to have high efficiency in heterogeneous catalysis in numerous reaction systems.^64,65^

The results of slight reduction in iron content from molybdenite concentrate show that changes in the morphology of molybdenum trioxide after oxidation roasting are controlled by copper amount in the form of chalcopyrite.^66^

**Fig. 8:**
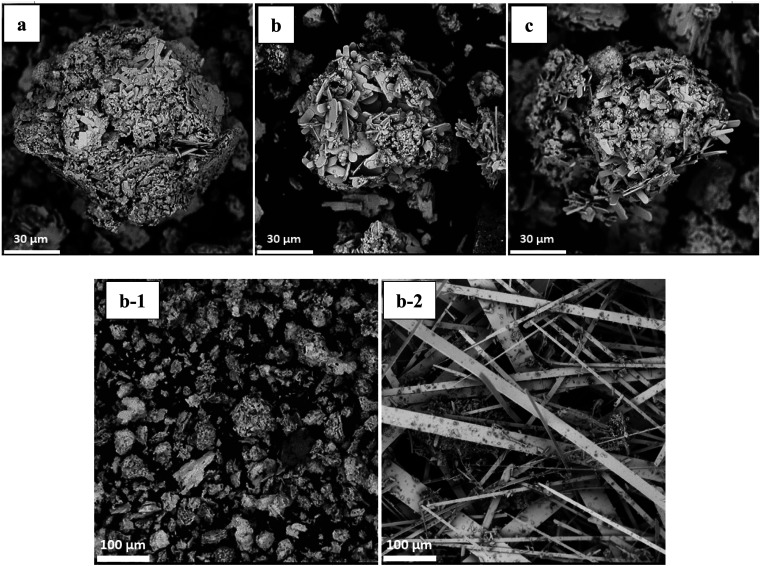
SEM images of roasted impure MoO_3_ at (a) 600 °C; (b) 625 °C; (c) 650 °C. (b-1) roasted impure and (b-2) roasted purified MoO_3_ at 625 °C.

To obtain the optimum conditions for the oxidative roasting process, a series of experiments were conducted using the roasting temperature and the process time as main factors. SEM images (Fig. 8) show that the size reduction of the particles is a dominant phenomenon during the conversion of MoS_2_ to MoO_3_. Furthermore, the apparent surface of particles formed is that of quasi-hexagonal multilayer sheets, while the formation of short fibers is seen after the roasting process. Generally, MoO_3_ fibers have a small surface area as a consequence of the oxidative roasting reaction of molybdenite.

The oxidation process of MoS_2_ can occur inside an electrical furnace in presence of atmospheric oxygen. Molybdenum trioxide, which is converted in two stages as the reaction product between MoS_2_ and O_2_, is produced (MoS_2_ → MoO_2_ → MoO_3_), according to the reactions (8) and (9):^67,68^

8 MoS_2_ + 3O_2_ → MoO_2_ + 2.5SO_2_

9 MoO_2_ + 0.5O_2_ → MoO_3_

The overall reaction (10) of the oxidation process to convert molybdenum disulfide to trioxide can be written as follows:^68^

10 MoS_2_ + 3.5O_2_ → MoO_3_ + 2SO_2_

It appears that the conversion rate to molybdenum trioxide is relatively low and occurs incompletely below 500 °C but can be accelerated at higher temperatures although there is a propensity for sintering above 625 °C owing to the presence of copper in the chemical composition of the concentrate.^69^ One of the reasons for sintering is the fairly low melting point of MoO_3_ which occurs at about 795 °C with a reaction between copper and MoO_3_ that lowers the melting point of the latter and accelerates the sintering of the fibers.^70^ Low-grade molybdenite concentrate has a copper content of 2.54% (in the form of chalcopyrite) compared to purified molybdenite with a copper content around 0.10%. Thus, sintering of impure concentrate could be caused by copper oxide and molybdenum trioxide forming a eutectic at 700 °C so, it is likely that significant sintering could take place at temperatures above 600 °C. At above 620 °C, the phenomena of melting/glazing simultaneously start to take place.^71^

In this research, particles of low grade molybdenite concentrate were investigated and the overall shape change of all the molybdenite particles after the oxidative roasting was considerable, as indirectly verified by scanning electron microscopy (SEM) as shown in Fig. 8. It appears that 625 °C is the best temperature for achieving more uniform fibers (Fig. 8b) as their growth is incomplete at lower temperature (600 °C) (Fig. 8a). However, agglomeration and sintering happen at higher temperatures (650 °C) (Fig. 8c) owing to the presence of copper that acts as an obstacle for the crystalline growth of molybdenum trioxide fibers. The SEM images of the molybdenum trioxide powder obtained after the purification of molybdenite concentrate exhibited a size change of fibers morphologically. The initial particles in the form of quasi-hexagonal molybdenum disulfide flakes have been predominantly changed into quasi-rectangular fiber structures after heating at 625 °C for 1 h. The size of the purified rectangular fibers is larger than the fibers produced from impure molybdenite concentrate. However, when the roasting temperatures are higher than 625 °C, the impure-MoO_3_ fibers joined together and sintering occurred. Additionally, it is evident that the agglomerated structure of the high-chalcopyrite MoO_3_ fibers decreases with increasing purity of the molybdenite concentrate used.

The obtained results confirm that iron removal, almost completely, can be led to the formation of thinner fibers with a more uniform diameter.^40–42^ While, after leaching of molybdenite under the designed conditions, the removed iron impurity was measured around 40 to 50% that it may be considered in the form of CuFeS_2_. In the other words, Iron in the form of FeS_2_ was not dissolved in the acid solution or its dissolution amounts was trace. Nonetheless, using nitric acid have eliminated the highest content of Cu, over 95%, from molybdenite concentrate. So, according to the research findings, the presence or absence of copper in molybdenite concentrate composition is only affected parameter that plays a critical role in increasing the length of MoO_3_ fibers. It can, therefore, be deduced that changes of molybdenite composition have a direct impact on the MoO_3_ morphology.

The results show that the morphology of the molybdenum trioxide produced is controlled by the copper content of the molybdenite concentrate used and that is influenced by of the purification conditions and temperature of the leaching process. Thus, copper in the form of chalcopyrite plays a negative role in the crystalline growth of MoO_3_ fibers as their length can be considerably increased after decreasing the copper content.

Fig. 8(b) and (b1) illustrates that the clustered fibers have a short coral-like fiber morphology with mean diameters and lengths of about 3 and 20 μm, respectively. From the high magnification SEM image of impure MoO_3_, it can be seen that the micro-clusters are constructed by rather rough microfibers with the average diameters of about 150–200 nm, which seemingly have grown around a center. In order to survey the growth mechanism and control the morphology of MoO_3_ microfibers, some experiments have been performed including purification and copper reduction by leaching process and optimization of roasting temperature. Different morphologies of MoO_3_ fibers (shown in Fig. 8(b2)) can be obtained after leaching by nitric acid, implying that chalcopyrite reduction plays an important role in the control of the morphology of MoO_3_ fibers. After purification *via* HNO_3_ leaching and calcination at 625 °C in air for 1 h, the coral-like morphology of the MoO_3_ almost disappeared.

**Fig. 9:**
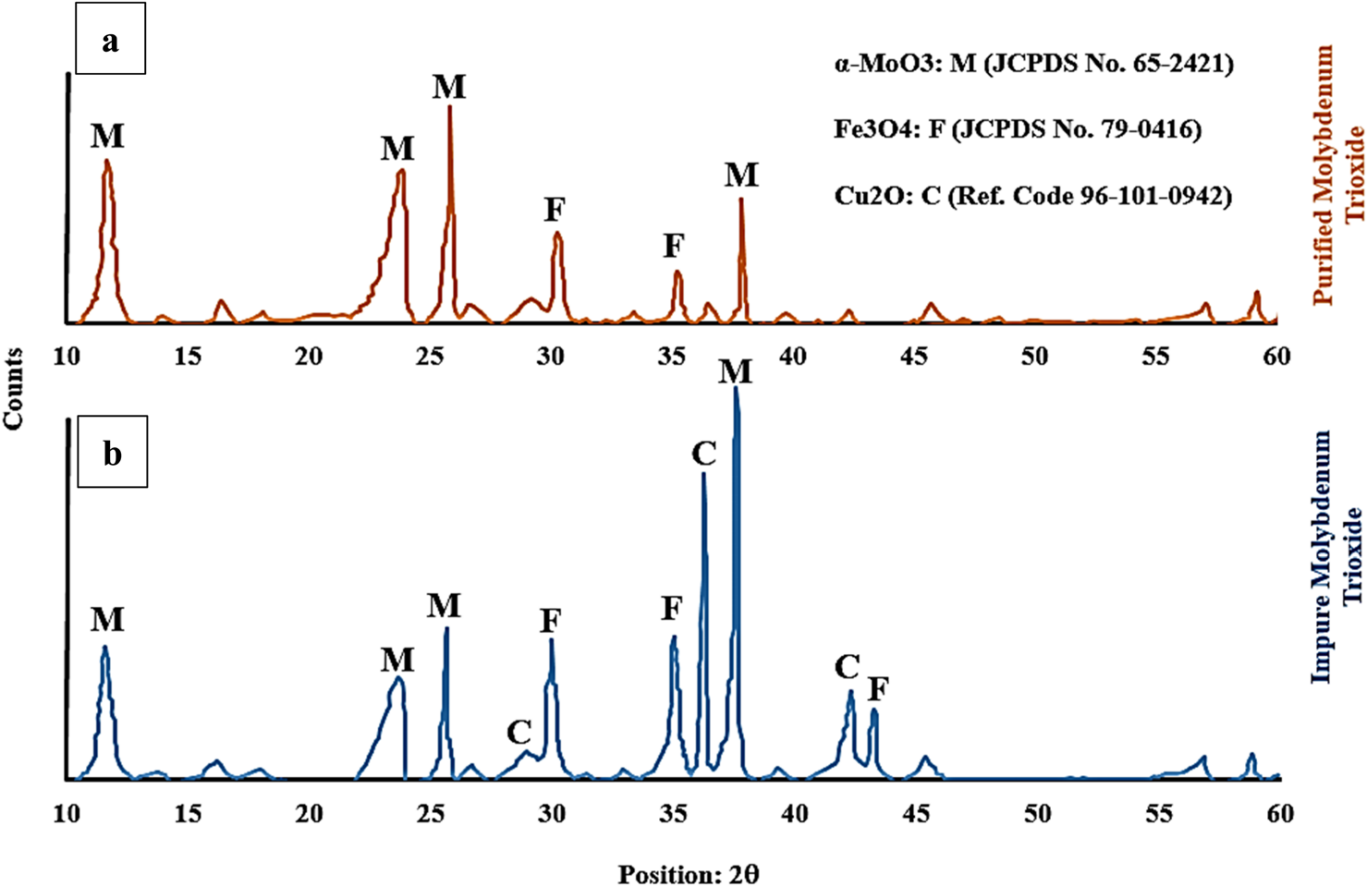
The XRD graph of impure and purified molybdenum trioxide.

Fig. 9 shows the XRD graph to identify the impurities present in the purified (Fig. 9a) and impure (Fig. 9b) molybdenum trioxide after roasting in air atmosphere. The phase transformation temperature of chalcopyrite to its oxide state depends on various factors such as heating rate, oxygen pressure and the purity of the chalcopyrite. The phase transformation of chalcopyrite (between 500 °C to 800 °C) and pyrite (between 500 °C to 900 °C) to their oxide state results in the formation of copper and iron oxides. At mentioned temperatures, pyrite decomposes to form iron oxide (FeO) and sulfur dioxide (SO_2_). The main copper oxide formed during this process is cuprous oxide (Cu_2_O) while the iron oxide produced is mainly magnetite (Fe_3_O_4_). However, other iron oxide phases such as hematite (Fe_2_O_3_) and wustite (FeO) may also be produced depending on reaction conditions. Mineralogical investigation of the samples (IMT and PMT) showed α-MoO_3_ as the main oxide that is also known as a catalytic phase as well as Cu_2_O and Fe_3_O_4_ as the minor mineral impurities present.

The crystalline α-MoO_3_ structure was indexed at 12.3°, 23.3°, 26.4°, and 38.7° corresponding to the (020), (110), (040), and (060) crystal planes, which matched with the corresponding standard card (JCPDS card No. 65-2421) of the catalytic MoO_3_ structure.

Additionally, the X-ray diffraction (XRD) pattern for pyrite present in molybdenite may vary depending on the specific sample and the experimental conditions used in the study. The standard card of JCPDS card No. 79-0416 indicates that characteristic peaks for magnetite are typically observed at around 30.1°, 35.6°, and 43.2° which correspond to the (220), (311), and (400) crystallographic planes, respectively.

The XRD pattern for Cu_2_O, as the reference code 96-101-0942, has peak positions of 28.5°, 35.5°, and 38.7°, which correspond to the (110), (111), and (200) crystallographic planes, respectively. The successful purification of the molybdenite concentrate through the HNO_3_ leaching process is confirmed by the absence of a cuprous oxide peak in Fig. 9a.

## 4. Conclusions

Removing impurities, especially chalcopyrite, from low-grade molybdenite concentrate (RMC) in nitric acid media was studied using response surface methodology. The effect of operating factors including reaction time, leaching temperature, and HNO_3_ molarity were addressed by central composite design. It was observed that copper in the form of chalcopyrite as the main impurity declined considerably (more than 95%) under optimum conditions. According to the results of these experiments, to maximize copper removal, a reaction time of 2 h, reaction temperature of higher than 80 °C, HNO_3_ molarity of 0.6 M, pulp density of 40 g L^−1^ (32 g raw molybdenite: 800 mL HNO_3_ solution) and same rotation speed of 500 rpm should be selected. The morphology of the molybdenum trioxide is mainly investigated by SEM images with the conclusion copper acts as an obstacle to the crystalline growth of molybdenum trioxide fibers. The results showed that the removal of copper impurity from molybdenite is only positive effective factor on enhancement of fiber length. Meanwhile, the presence or absence of iron impurities, in the forms of CuFeS_2_ and FeS_2_, are ineffective on the further growth of molybdenum trioxide fibers. Thus, chalcopyrite elimination resulting from molybdenite leaching, plays an important role in controlling the morphology of MoO_3_ and enhancing the length of quasi-rectangular microfibers from less than 30 μm for impure IMT up to several centimeters for PMT.

## Conflicts of interest

The authors declare that they have no competing interests.

## Abbreviations

CCD: Central composite design
ICP-MS: Inductively coupled plasma-mass spectrometer
ICP-OES: Inductively coupled plasma-optical emission spectrometry
IMT: Impure molybdenum trioxide
PMC: Purified molybdenite concentrate
PMT: Purified molybdenum trioxide
RMC: Raw molybdenite concentrate
RSM: Response surface methodology
SEM: Scanning electron microscope
WMC: Washed molybdenite concentrate
XRD: X-ray diffraction

## Supplementary Material

RA-013-D3RA02384B-s001
