# Supplementary figures and images for: Investigation of chalcopyrite removal from low-grade molybdenite using response surface methodology and its effect on molybdenum trioxide morphology by roasting
Source: RSC Adv. 2023 May 15;13(22):14899–913. doi: 10.1039/d3ra02384b (PMC10184750; doi:10.1039/d3ra02384b)

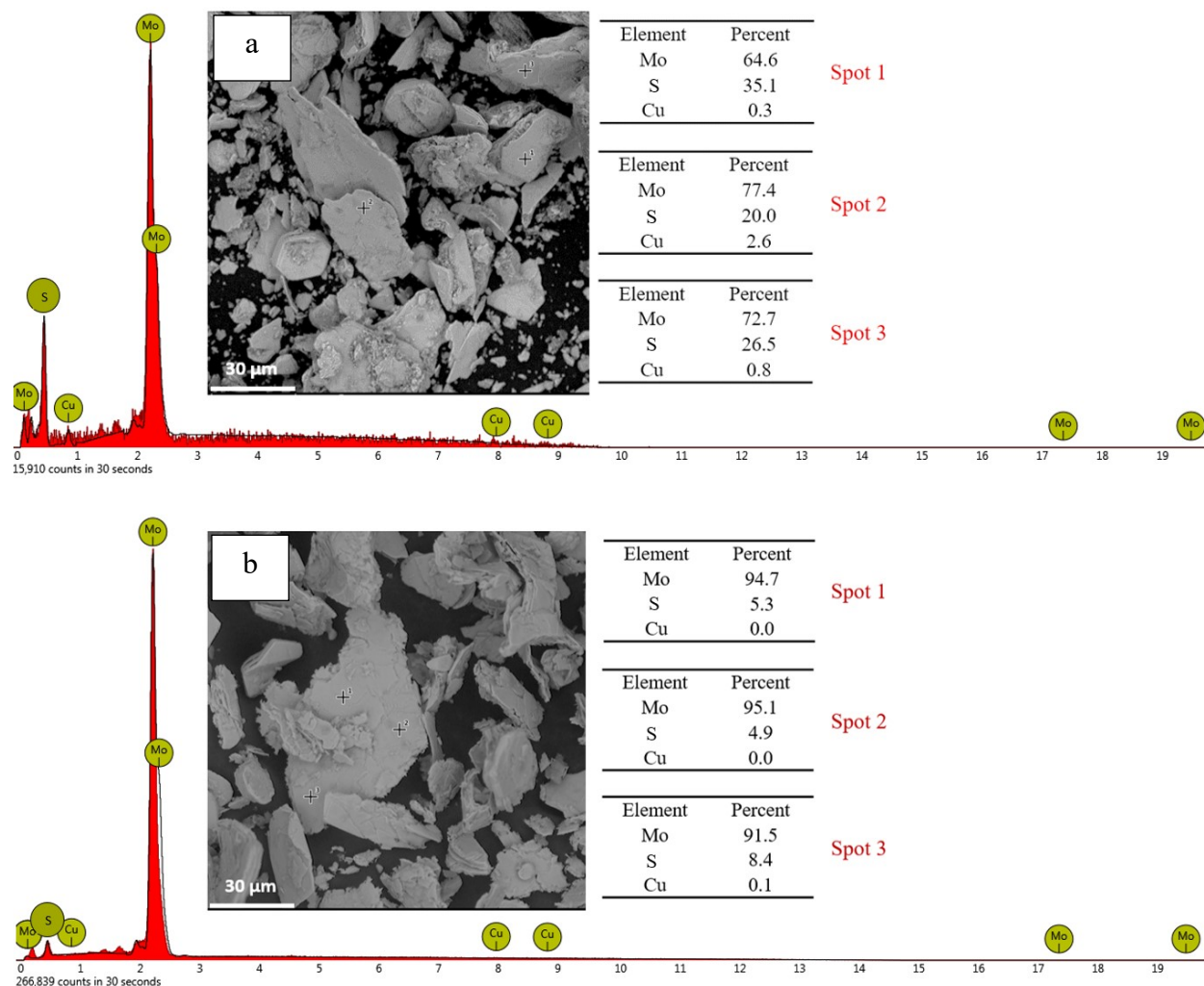

Figure S1. Figure 1. EDS analysis of (a) impure and (b) purified molybdenite concentrate.

Supplement: RA-013-D3RA02384B-s001 [file RA-013-D3RA02384B-s001.pdf]
